# Mental Health and Subjective Vitality in Arabic-Speaking North Africa: A Cross-Cultural Examination of the Mental Health Continuum–Short Form (MHC-SF)

**DOI:** 10.1007/s11126-025-10173-3

**Published:** 2025-06-27

**Authors:** Rasha Mohamed Abdelrahman, Walid Ahmed Massoud, Marei Ahmed

**Affiliations:** 1 Psychology Department, College of Humanities and Sciences, Ajman University, Ajman, UAE; 2 Department of Psychological Sciences, College of Education, Qatar University, Doha, Qatar; 3 National Center for Examinations and Educational Evaluation (NCEEE), Cario, Egypt

**Keywords:** Mental Health Continuum-Short Form, Subjective Vitality, Measurement Invariance, Arabic-speaking populations

## Abstract

This study aimed to assess the validity of the Mental Health Continuum-Short Form (MHC-SF) as a measure of mental health among Arabic-speaking North African populations. The study had three objectives: (1) to evaluate the structural validity of the MHC-SF and the Subjective Vitality Scale (SVS); (2) to examine measurement invariance across gender and between Moroccan and Egyptian participants; and (3) to assess whether emotional, social, and psychological well-being dimensions predict subjective vitality. A total of 301 psychology students were recruited from Morocco and Egypt. Using CFA, a three-factor model for the MHC-SF demonstrated good structural validity and the SVS showed good fit, as well. Significant correlations between subjective vitality and mental health were observed, and the regression analysis revealed that mental health factors predicted subjective vitality. The findings support the use of the MHC-SF in Arabic-speaking populations and underscore the relevance of cultural context in assessing mental health.

## Introduction

The emergence of positive psychology has led to new mental health approaches. According to the World Health Organization's latest definition (WHO)[51], mental health is described as"a state of well-being in which every individual realizes their own potential, can cope with life's normal stresses, can work productively and fruitfully, and can contribute to their community."

These perspectives highlight the importance of positive functioning, meaningful objectives, satisfying activities, and optimistic growth in mental well-being. As a result, the components of positive mental health and the symptoms of mental disorders can evolve. This viewpoint considers an individual's strengths and weaknesses and recognizes that a comprehensive mental state assessment requires integration.

The understanding of the connection between mental health and mental disorders has recently changed, impacting the conceptualization of mental health. Previously, mental health was defined by the WHO as the absence of mental illness [6]. However, focusing only on the prevention and therapy of mental disorders has not effectively reduced its prevalence [10]. Weisz et al. [48] demonstrated that psychological interventions for children and adolescents over the past 50 years have not resulted in significant improvement.

Several models have been anticipated to conceptualize and evaluate mental health. Keyes’ theory [19] acknowledges that individuals are part of social structures, confront diverse social challenges, and engage in various interpersonal interactions, thereby considering the social dimension of mental health. Keyes [19] developed two different but interconnected continuum models rather than a single continuum, with mental well-being and disorders at opposite ends. This Mental Health Continuum (MHC) categorizes positive mental health into three levels: flourishing, moderate, and languishing [23]. The MHC theoretical model divides mental health into three factors: emotional (EW), social (SW), and psychological (PW) well-being.

Emotional well-being encompasses both positive emotions and life satisfaction [3]. Psychological well-being includes components of individuals'psychological functioning such as autonomy and personal growth [38]. Social well-being emphasizes individuals'judgments of their social lives, reflecting their appraisal of their conditions and societal functioning [19]. According to Keyes [19], a comprehensive perspective that encompasses emotional, psychological, and social well-being is necessary for achieving mental health. He defined flourishing as a state in which individuals experience a high level of subjective well-being, coupled with optimal psychological and social functioning.

In the same way, he defined languishing as a state in which individuals have low subjective well-being, as well as low psychological and social well-being. People with average mental health status are neither declining nor flourishing. The identification of low subjective well-being corresponds to the criteria for depression as specified in the DSM-V. This includes experiencing feelings of anhedonia, such as sadness, loss of interest, and diminished pleasure, as well as encountering functional problems, such as issues with appetite, sleep, or fatigue [19]. Individuals who have flourishing mental health tend to display enjoyable and positive performance, as opposed to those with languishing mental health, who experience low levels of pleasure [20]. According to epidemiological research, MHC has been linked to improved physical, mental, and psychosocial functioning.

The Mental Health Continuum-Short Form (MHC-SF) is a self-report measure that has increased in use for assessing well-being at the population level [22, 49]. Keyes developed an efficient version of his original Mental Health Continuum-Long form for epidemiological surveillance [19]. The MHC-SF encompasses both hedonism and eudaimonism, the dominant well-being traditions in literature. Its items and components align well with the widely recognized and cited definition of mental health by the WHO, which highlights well-being as the realization of one's abilities, effective coping with life stressors, productive and fruitful work, and contributions to the community [51]. This definition of mental health encompasses both hedonic and eudaimonic well-being, and takes into consideration the psychological and social components of human functioning. The MHC-SF has been widely used in research due to its well-established psychometric properties. However, there remains a need to validate this scale in underrepresented cultural contexts, such as Arabic-speaking populations in North Africa, where well-being may be conceptualized variously due to religious, social, and communal norms. This study addresses this gap by assessing the reliability and structure of the MHC-SF in two culturally distinct Arab nations.

By exploring how participants from Morocco and Egypt interpret and respond to the MHC-SF, we contribute to the applicability of this instrument globally, and examine whether cultural differences influence the understanding of emotional, social, and psychological well-being.

Various researchers have employed confirmatory factor analysis (CFA) to assess the structural validity of the MHC-SF and evaluate model fit [8, 26, 31, 34]. However, relying solely on this approach is limited when dealing with measures that exhibit multi-dimensional item responses [35]. First, the interpretation of the total scale score as a single construct may be compromised by its multidimensionality, which is often overlooked. Second, previous studies have not clearly established the extent to which subscale scores are justified. If so, the informational value of the subscales, beyond the total scale score, remains uncertain. Consequently, some researchers argue that the proposed correlated three-factor structure of the MHC-SF, initially suggested by Keyes, poses challenges both theoretically [2] and empirically as it often yields only marginally acceptable fit indices. and from empirical side as it often yields only marginally acceptable fit indices [17].

The factor structure of the MHC-SF has been thoroughly examined using various techniques including exploratory factor analysis. (EFA)[26], confirmatory factor analysis (CFA)[8, 34, 40] and exploratory structural equation modeling (ESEM) [13]. Studies have revealed that the three-factor model (including emotional, social, and psychological well-being as factors) tends to fit better than the one-factor (mental health), two-factor (hedonic and eudaimonic well-being), or second-order factor models (consisting of three correlated first-order factors that reflect emotional, social, and psychological well-being, all of which are loaded into a second-order general well-being factor).

Research studies have consistently shown that the three-factor model of the MHC-SF exhibits adequate internal consistency and test–retest reliability [24, 26, 30, 34]. The Cronbach's coefficient alpha for both the general and subscale scores in the bifactor model was deemed suitable across studies, as well, [2, 5]. Despite using McDonald's ω reliability coefficient to evaluate the reliability of the scale scores in the bifactor model, the outcomes were not as conclusive. Several studies have indicated good reliability of the scores for the general and single well-being subscales based on ω coefficients [25, 36], while others have suggested that the general factor plays a more significant role in explaining the variance found in the MHC-SF scores. Furthermore, the three subscales demonstrated low reliability and explained minimal variance compared to that was explained by the general factor [5, 17, 28, 42–44].

According to the literature, the measurement invariance of the MHC-SF has been extensively examined, with considerable evidence supporting the invariance of its structure across different groups, contexts, and conditions. Most studies observed measurement invariance across gender and age for both the three-factor and bifactor solutions of the MHC-SF [4, 8, 13–15, 25, 30]. Lamers et al. [26] examined the longitudinal measurement invariance of the bifactor model and found that there is no differential item functioning over time. Furthermore, the evidence suggests that MHC-SF demonstrates either full or partial cross-cultural measurement invariance. According to Joshanloo [12], the items of the MHC-SF performed similarly in the Iranian and American samples when employing the three-factor model. Similarly, Schutte and Wissing [43] found partial invariance in the bifactor model over three South African cultural groups. Moreover, Zemojtel-Piotrowska et al. [36] confirmed the cross-cultural measurement invariance of the MHC-SF bifactor structure across 38 cultures.

## Research Problem

The MHC–SF has been widely adopted by many countries, and substantial evidence supports its usefulness, validity, and reliability. Several studies conducted in various countries, including South Korea, Italy, Poland, South Africa, and France, have confirmed the three-factor structure of emotional, psychological, and social well-being previously identified in the United States. For example, Joshanloo and his colleagues [12, 16] investigated the MHC–SF across cultural groups from the Netherlands, South Africa, and Iran, and found consistent support for the three-dimensional structure. Moreover, the MHC–SF has demonstrated strong internal consistency and criterion validity in adolescents and adults from countries such as the United States, Netherlands, Egypt, Italy, and South Africa. Petrillo et al. [34] discovered that MHC subscales were positively correlated with corresponding measures of functioning and well-being, providing evidence of convergent validity. In addition, many studies showed that mental health positively correlates with subjective vitality in different samples and cultures [7, 29, 39, 40]. The results only showed a positive correlation between the two scales. No study has explored positive mental health (EW, SW, and PW) as a predictor of perceived vitality. In this study, we examined whether subjective vitality can be predicted by positive mental health.

In the present research, we have three objectives. The first one aimed to test 1) the reliability and structural validity of both the MHC-SF and the Subjective Vitality Scale (SVS) in the Arabic population; 2) the measurement invariance between gender (males and females) and nationality (Morocco and Egyptian); and 3) the prediction of MHC-SF subscales for subjective vitality.

## Material and Methods

### Participants

The sample consisted of 301 undergraduate and graduate psychology students purposefully selected from two Arab countries (Morocco and Egypt). The first country represents Northwest Africa (54 males and 160 females), and the second represents Northeast Africa (8 males and 79 females). The sample was 18–64 years (M = 30.1, SD = 12.65). A detailed summary of the sample's gender, marital status, education, and income is provided in Table 1 to enhance understanding of the participants'sociodemographic background.

**Table 1:** Sociodemographic Characteristics of Participants

Characteristic	Morocco (n = 214)	Egypt (n = 87)	Total (n = 301)
**Gender**			
Female	160 (74.8%)	79 (90.8%)	239 (79.4%)
Male	54 (25.2%)	8 (9.2%)	62 (20.6%)
**Marital Status**			
Single	94 (43.9%)	76 (87.4%)	170 (56.5%)
Married/Partnered	95 (44.4%)	5 (5.7%)	100 (33.2%)
Divorced/Widowed	25 (11.7%)	6 (6.9%)	31 (10.3%)
**Education Level**			
Postgraduate	89 (41.6%)	2 (2.3%)	91 (30.2%)
B.Sc. Degree	120 (56.1%)	79 (90.8%)	199 (66.1%)
High School	3 (1.4%)	6 (6.9%)	9 (3.0%)
Middle School	2 (0.9%)	0 (0.0%)	2 (0.7%)
**Monthly Income (USD)**			
> $3,000	1 (0.5%)	0 (0.0%)	1 (0.3%)
$2,400–2,999	3 (1.4%)	1 (1.1%)	4 (1.3%)
$1,800–2,399	6 (2.8%)	0 (0.0%)	6 (2.0%)
$1,200–1,799	16 (7.5%)	3 (3.5%)	19 (6.3%)
$600–1,199	67 (31.3%)	4 (4.6%)	71 (23.6%)
< $600	121 (56.5%)	79 (90.8%)	200 (66.5%)

## Procedures

Participants were recruited through their academic institutions, and data collection took place in classroom settings and university labs. Prior to participation, informed consent was obtained, and the study purpose was clearly explained. We emphasized the anonymity and confidentiality of the responses. Each participant completed the questionnaire packet, which included the MHC-SF and SVS, in approximately 15–20 min under the supervision of a research assistant. Ethical approval was obtained according to the guidelines of Ajman University, Research Ethics Committee at the University Deanship of Graduate Studies and Research (Reference number: H-F–H-20-Mar). The instruments were administered in Arabic language. The Arabic versions of the scales had been validated in prior studies, ensuring linguistic and cultural appropriateness.

## Instruments

### The Mental Health Continuum-Short Form (MHC-SF)

 [24] is a concise questionnaire that assesses positive mental health. It consists of 14 items that capture various aspects of well-being. Specifically, three items evaluated emotional well-being (items 1–3) (for example, How frequently did you feel happy in the past month), five items related to social well-being (items 4–8) (for example, How frequently did you feel that you had something important to contribute to society in the past month), and six items addressed psychological well-being (items 9–14) (for example, How frequently did you feel that you liked most parts of your personality in the past month). Participants rated the frequency of experiencing each feeling over the past month on a 6-point Likert scale, ranging from 0 (never) to 5 (every day). MHC-SF serves two purposes. First, it assesses three dimensions of well-being: emotional, social, and psychological well-being. Second, it enables a categorical diagnosis of mental health status by classifying individuals as flourishing, moderate, or languishing. This study used an Arabic version, which was translated and validated in previous studies [11, 39].

### Subjective Vitality Scale (SVS)

According to Ryan and Frederick [37], the SVS is a short instrument for measuring vitality. A 7-point Likert scale was used, ranging from"strongly disagree"(1-point) to"strongly agree"(7-point). We used the Arabic version validated by Salama-Younes [39] in an Egyptian population. This study used a 5-point Likert scale that is more adaptable to the Arabic population. Experts in content validity have confirmed this finding. It ranges from 1 (disagree) to 5 (agree). In the present study, we used a version containing six items validated for adolescent athletes [39, 41].

## Statistical Analysis

Descriptive statistics were used to explore the score distribution. The sub-scores of emotional well-being (items 1–3; range 0–15), social well-being (items 4–8; range 0–25), and psychological well-being (items 9–14; range 0–30) were obtained by summing the relevant subscale items. The overall score of well-being was calculated by summing the 14 items of MHC-SF (range: 0–70). The diagnostic categories proposed by Keyes [21] were used to estimate the prevalence of mental health categories in the population. For a diagnosis of flourishing, individuals must experience one of the three hedonic well-being symptoms (items 1–3)"every day"or"almost every day"and six of the eleven positive functioning symptoms (items 4–14)"every day"or"almost every day"in the past month. Languishing is diagnosed if an individual reports feeling the three hedonic well-being symptoms"never"or"once or twice"and six of the eleven eudaimonic well-being symptoms"never"or"once or twice"in the past month. Individuals who do not meet the criteria for either"languishing"or"flourishing"are diagnosed as"moderately mentally healthy."

A Confirmatory Factor Analysis (CFA) was carried out using IBM AMOS 22 to assess the structural validity of the Arabic version of the MHC-SF. This was done by testing three distinct models based on theoretical foundations and prior research, as described in the Introduction. These models included: (1) a single-factor model that loaded all 14 items onto a general well-being factor, (2) a two-factor model consisting of two correlated factors: hedonic well-being (EWB, items 1–3) and eudaimonic well-being (SWB and PWB, items 4–14), and (3) a three-factor model consisting of three correlated factors of well-being (EWB, items 1–3; SWB, items 4–8; PWB, items 9–14).

Participants who had missing data were not included in the analysis. To assess the model's absolute fit, the Satorra-Bentler scaled chi-square test (SB χ2) was utilized. However, to ensure a thorough evaluation of the model's fit, additional absolute and incremental fit indices were employed. The absolute fit indices consisted of the ratio of SB χ2 to degrees of freedom (SB χ2/df), the Standardized Root Mean Square Residual (SRMR), the Root Mean Square Error of Approximation (RMSEA), and the Akaike's Information Criterion (AIC). The incremental fit indices included the Tucker-Lewis Index (TLI) and the Comparative Fit Index (CFI). A good model fit was indicated by values of SB χ2/df < 3, SRMR and RMSEA < 0.06, and TLI and CFI > 0.95, while a satisfactory fit was suggested by SB χ2/df < 5, SRMR and RMSEA < 0.08, and TLI and CFI > 0.90 [9]. In general, lower AIC values indicate a better fit.

## Results

### Reliability for MHC-SF and SVS

The reliability of the MHC-SF was evaluated using internal consistency and composite reliability measures. A value greater than 0.7 was considered acceptable [21]. The results of the analysis are presented separately for different factors. Descriptive statistics (means and standard deviations), Cronbach's alphas, and composite reliability coefficients for the latent MHC-SF and SVS are presented in Table 2.

**Table 2:** Internal Consistency and Reliability of MHC-SF Total Scale, 3 MHC Factors, and SVS Scale

Item no	MHC-SF				SVS	
	Total Score		3 Factors Scores		Total Score	
	Corrected Item-Total Correlation		Corrected Item-Total Correlation		Corrected Item-Total Correlation	
1	0.642		0.751		0.751	
2	0.642		0.738		0.313	
3	0.571		0.574		0.754	
4	0.550		0.425		0.738	
5	0.573		0.512		0.809	
6	0.594		0.553		0.807	
7	0.454		0.471			
8	0.430		0.402			
9	0.524		0.503			
10	0.661		0.681			
11	0.693		0.586			
12	0.534		0.620			
13	0.573		0.643			
14	0.628		0.612			
**EWB 1 Alpha**			**0.826**			
**EWB 1 Omega**			**0.837**			
**SWB 2 Alpha**			**0.715**			
**SWB 2 Omega**			**0.720**			
**PWB 3 Alpha**			**0.833**			
**PWB 3 Omega**			**0.720**			
**Total Alpha**	**0.891**				**0.878**	
**Total Omega**	**0.892**				**0.887**	
**Mean (SD)**	**62.55 (15.42)**		**13.17 (4.29)** **20.66 (6.49)** **28.72 (6.95)**		**20.00 (6.41)**	

## Structure Validity

**Fig. 1:**
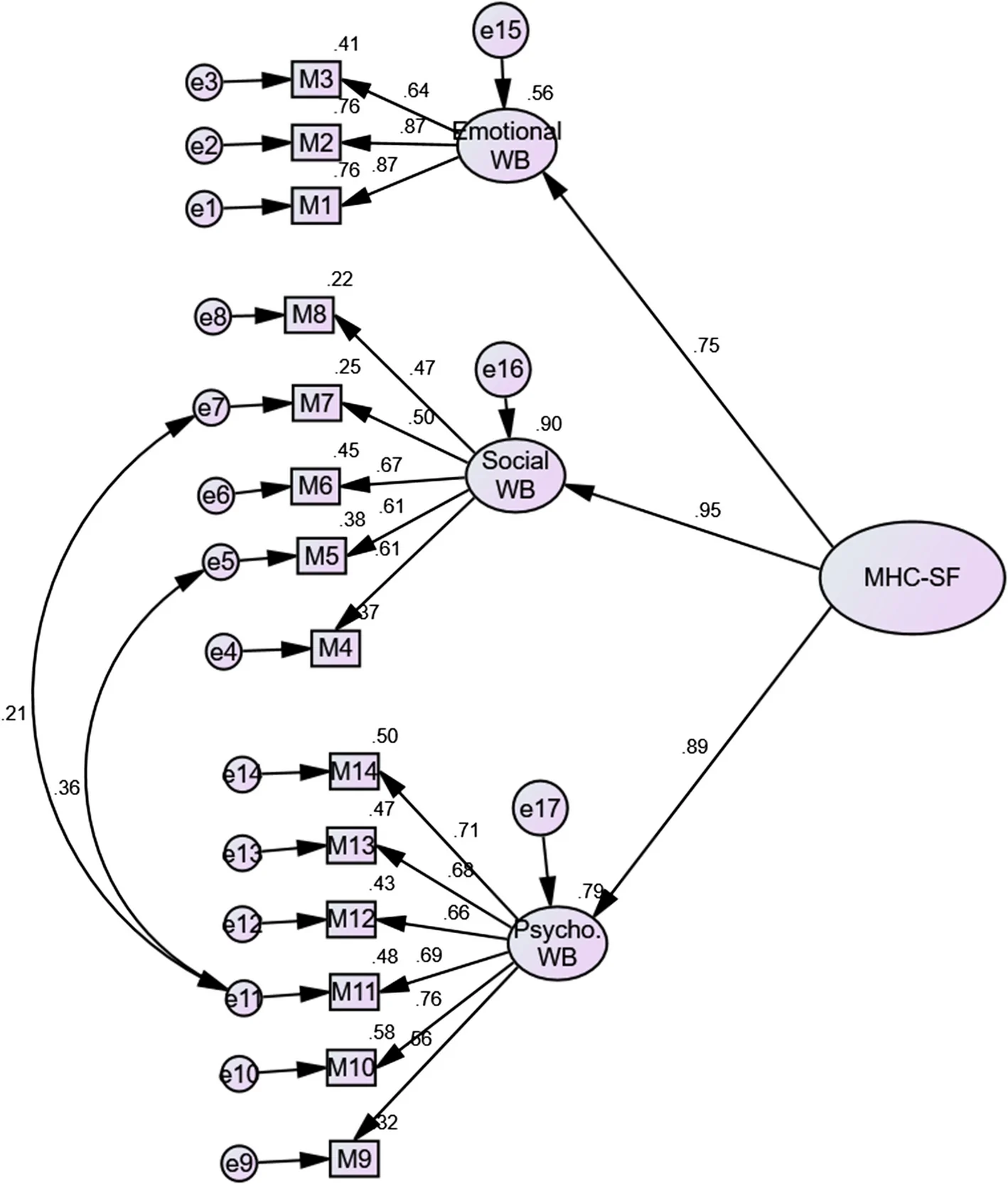
Factor Structure Model of MHC-SF (3 Factors Model)

We evaluated the one-factor, two-factor, and three-factor Confirmatory Factor Analysis (CFA) models, and their respective fit indices are presented in Table 3. The three-factor model was the only one that provided a good fit, as demonstrated in the AMOS v.22 input diagram in Fig. 1. On the other hand, the single-factor model had the lowest fit, followed by the two-factor correlated models, all of which failed to show an overall acceptable fit. The degrees of freedom (*df*), root mean square error of approximation (RMSEA), comparative fit index (CFI), and standardized root mean square residual (SRMR) were used to assess reliability and the percentage of common variance attributed to the general factor as an index of unidimensionality.

**Table 3:** Model Fit for MHC-SF and SVS, and MHC-SF Measurement Invariance across Gender and Nationality

Model	χ2 (df)	CMIN/df	CFI	IFI	TLI	RMSEA	SRMR	AIC	BIC
MHC-SF									
*All Samples*									
One factor model	411.601 (77)	5.345	0.822		0.767	0.120	0.073	467.601	571.400
Two-factor model	268.902 (76)	3.538	0.886		0.864	0.092	0.066	326.902	434.408
Three-factor models	207.214 (72)	2.878	0.920		0.900	0.079	0.061	273.214	395.548
*Gender*									
Configural Invariance	329.193 (144)		0.897	0.899	0.870	0.066	0.076		
Weak/Metric Invariance	343.475 (155)		0.895	0.897	0.877	0.064	0.090		
Strong/Scalar Invariance	375.936 (169)		0.885	0.885	0.876	0.064	0.092		
*Nationality*									
Configural Invariance	310.479	144	0.905	0.907	0.880	0.062	0.059		
Weak/Metric Invariance	333.248	155	0.899	0.900	0.881	0.062	0.063		
Strong/Scalar Invariance	387.026	169	0.876	0.877	0.866	0.066	0.064		
SVS									
One factor	23.525 (9)	2.614	0.987		0.978	0.073	0.023	47.525	92.010

*All Chi squares* were significant (*p* < 0.001)

To avoid redundancy, descriptions of the scales previously included in the Methods section were not repeated here. Instead, the focus remains on the statistical outcomes. Explanatory footnotes have been added to all tables to define abbreviations such as CFI, RMSEA, and SRMR.

### Results of CFA for MHC-SF and SVS

Table 4 presents the fit indices of the three-factor model. It shows RMSEA (0.079) under 0.08, SRMR (0.061) under 0.08, CFI (0.920) above 0.95, and TLI (0.900) = 0.90, indicating that the sample correlation matrix is well recovered, i.e., *df* = degrees of freedom, RMSEA = root mean square error of approximation, SRMR = standardized root-mean square residual reliability, and, CFI = comparative fit index. SVS was analyzed using the same method.

**Table 4:** Correlation and Regression of SVS on MHC-SF

MHC-SF factors	Pearson Correlation	Adjusted R Square	F	Unstandardized Coefficients		Standardized Beta	t
				B	Std. Error		
*(Constant)*				*0.168*	*0.184*		*0.910*
Emotional Well-being	0.579**	0.524	111.299**	0.156	0.039	0.209	4.018**
Social Well-being	0.639**			0.236	0.047	0.287	5.058**
Psychological Well-being	0.660**			0.314	0.053	341	5.904**

*p < 0.05. **p < 0.01. ***p < 0.001; *Note. Dependent variable: SVS*

The MHC-SF aims to measure both the hedonic and eudaimonic aspects of mental well-being, and a strong correlation is expected. Using the AMOS models shown in Fig. 1, the General Well-being (GW) factor of the MHC-SF was correlated with a corresponding GW factor identified for SWEMWBS. This correlation was initially determined through Exploratory Factor Analysis (EFA) and subsequently tested using a three-factor Confirmatory Factor Analysis (CFA) model, which demonstrated an acceptable-to-good fit (RMSEA = 0.079; CFI = 0.920; SRMR = 0.061). A correlation coefficient of 0.86 was found between the two modeled GW factors. Concerning the SVS, the goodness of fit is acceptable for SVS was also acceptable (RMSEA = 0.073, CFI = 0.987, SRMR = 0.023). The results show that the two scales have adequate structural validity.

## Measurement Invariance

The present study sample comprised 62 (20.6%) males and 239 (79.4%) females. There were 87 (28.9%) Egyptians and 214 (71.1%) Moroccan. In the first step, we examined the assessment of normality for the male, female, and invariance indices. We then examined the normality between the two nationalities.

To investigate measurement invariance, multi-group analyses were conducted based on gender and nationality. The fit indices of different models were compared, explicitly comparing the fit indices of the model with constrained loading parameters (Metric Invariance) to the model with constrained structural parameters (Configural Invariance) and the fit indices of the model with constrained intercepts and measurement residuals (Scalar Invariance) to the model with constrained structural parameters (configural). Overall, as presented in Table 3, there were either no changes or only slight changes in the fit between the models, suggesting measurement invariance. The CFI (Comparative Fit Index) was the only fit measure that showed a significant decrease when comparing the scalar and configural invariance models (ΔCFI = 0.012).

### Measurement Invariance for Gender

For the male cohort, Mardia's coefficient was calculated to be 93.029, with a critical ratio (CR) of 17.304, which exceeds the threshold of 1.96, indicating statistical significance. However, multivariate normality was not observed. In contrast, the female cohort exhibited a Mardia's coefficient of 51.787, with a CR of 18.912, also surpassing the 1.96 threshold, thereby confirming statistical significance. Thus, multivariate normality was not met, and bootstrapping was used to overcome this limitation.

The total sample's factor loadings did not show gender differences (Table 3), indicating metric or"weak"invariance across genders. This means that the latent variable is similarly associated with the items for both men and women. However, when further constraints were applied to equate the intercepts (strong invariance) and residuals (strict invariance), the *χ*2 test showed a significant decrease in the fit. It is worth noting that analyses based on large samples can result in higher *χ*2 values, which may lead to rejection of good models. In the present study, the additional fit indices improved (RMSEA) or slightly decreased (CFI) when constraints were added. This suggests that fixing the intercepts and residuals to equality in males and females supports the assumption of strict invariance across genders. This implies that the group means for the latent variable and analyses involving correlations with the latent variable can be compared across genders. These findings are consistent with those of previous studies.

### Measurement Invariance for Nationalities

Mardia's coefficients for males (93.029, CR = 17.304 > 1.96) and for females (51.787, CR = 18.912 > 1.96) were significant, and multivariate normality was not met. To overcome this limitation, we used the bootstrapping procedure. An analysis of measurement invariance was conducted across nationalities (Table 3). The invariance models demonstrated acceptable model fits, except for the strict factorial invariance model, in which the CFI and TLI values were lower than or equal to 0.90. RMSEA values were higher than 0.10. However, the robust factorial invariance model did not significantly differ in model fit compared with the configural invariance model, as indicated by the CFI and RMSEA values. Although MHC-SF did not exhibit strict invariance, it should be noted that strict factorial invariance is not a prerequisite for group comparisons of means and variances, as discussed by Byrne and Stewart [1] and Tse et al. [45]. According to Meredith and Teresi [33], it is essential to maintain the consistency of factor loadings among the examined groups for accurate comparisons of scale scores or latent variable means. While strict invariance may be less important in the context of basic scientific research, variations in specific factors among groups serve to highlight individual differences that are crucial for exploration and discovery. In addition, meeting the first three levels of invariance or achieving partial invariance is considered sufficient to guarantee appropriate cross-group comparisons of the latent constructs [46]. This means that the measurement invariance of the MHC-SF was partially upheld across nations. The observed differences in the latent means can be interpreted as genuine differences in the MHC-SF and are not attributable to psychometric issues. Consequently, the results obtained from both countries can be placed on the same international scale.

## Regression of Subjective Vitality on Mental Health

To achieve our third objective, we examined the correlations between subjective vitality and mental health scale factors. We found a significantly strong correlation between subjective vitality and mental health (*r* = 0.727; p < 0.001). In addition, all the correlations between subjective vitality and mental health factors were significant and larger than 0.50 and significant. Using linear regression, we could significantly predict subjective vitality from the factors of the mental health scale (adjusted R^2^ = 52%). These three factors were also significant, as shown in Table 4.

## Discussion

Due to the increased usage of well-being scales, conducting psychometric evaluations of these instruments is crucial to gain insights into how well-being and positive mental health concepts can be described and comprehended.

Hence, the first objective of the present research was to assess the reliability and structural validity of the MHC-SF in two different Arabic countries. Regarding reliability, the subscales and the total scale had adequate internal consistency. Furthermore, the study's results indicate that the MHC-SF's measure of positive mental health can be accurately represented by a three-factor model that predominantly includes a general well-being factor, along with three additional group factors that are specific to emotional, social, and psychological well-being. The three-model model exhibited the best goodness-of-fit. The SVS also had good validity structure and internal consistency.

Regarding the second objective concerning the measurement invariance, the factor loadings in the overall sample demonstrated no gender differences, indicating metric or"weak"invariance across genders. This implies that the latent variable is similarly associated with the items for both male and female participants. However, when further constraints were implemented to equalize the intercepts (strong invariance) and residuals (strict invariance), there was a significant decrease in the fit, according to the *χ*2 test. For the measurement invariance for nationality, the invariance models showed satisfactory model fits, except for the strict factorial invariance model, which had CFI and TLI values below 0.90 and an RMSEA value exceeding 0.10. Nevertheless, the strong factorial invariance model did not display substantial differences in model fit compared to the configural invariance model, as indicated by the CFI and RMSEA values. These findings align with previous studies conducted in other cultural contexts, such as Iran, South Africa, and Poland [12, 18, 43], where partial or full invariance of the MHC-SF was confirmed. Our results support the scale’s cross-cultural applicability while highlighting the cultural complexity in assessing well-being across Arab populations.

According to Wilson et al. [50], a systematic literature review found that 13 studies did not undertake a strict factorial invariance analysis but found weak or strong factorial invariance. Lau et al. [27] indicated that strict factorial invariance is not necessary to make mean comparisons among groups, but an assessment of scalar invariance is needed. Although the MHC-SF did not demonstrate strict invariance, it is essential to acknowledge that strict factorial invariance is not necessary for comparing group means and variances.

Concerning the third objective, the results showed a strong significant correlation between subjective vitality and mental health in general, which is consistent with the results of various studies [37, 39, 47] that have shown that subjective vitality is a dynamic reflection of well-being. In addition, the factors of mental health, that is, emotional, social, and psychological well-being, proved to be good predictors of subjective vitality.

These findings reflect how cultural conceptions of well-being may shape how participants respond to items on mental health scales. For instance, collectivist values commonly found in Moroccan and Egyptian societies may influence how social well-being is perceived and rated. This could explain the partial measurement invariance found across national groups.

## Conclusion

The MHC-SF is a psychometrically robust instrument that is used to assess the overall mental well-being of the Arabic population. Based on an extensive study involving this population, we found that MHC-SF is essentially unidimensional and can be best represented by a three-factor model. This model consists of a dominant general well-being factor and three specific group factors related to emotional, social, and psychological well-being. The general well-being factor demonstrated high internal reliability, whereas the subscales of the specific group factors exhibited adequate reliability. It is recommended not to rely solely on the subscales, as they are more reliable in measuring the general well-being factor than the specific group factors. The test–retest reliability of the MHC-SF was good, and convergent validity was supported.

The findings of this study have significant implications for mental health policy and care. Mental health care primarily focuses on psychopathology, which involves the diagnosis and treatment of mental disorders. Mental health and mental disorders are distinct aspects of mental well-being. Therefore, it would also be beneficial to emphasize the enhancement of positive mental health. This study aimed to pave the way for a more informed and comprehensive conceptualization and evaluation of mental well-being across various age groups, fostering a more inclusive approach.

The mental health initiatives in educational settings across North Africa could integrate MHC-SF screening tools culturally tailored to identify students with low levels of well-being and offer preventive interventions. This would be a cost-effective and culturally sensitive approach to promoting psychological wellness in universities and schools.

In this study, we used the Mental Health Continuum-Short Form (MHC-SF) developed by Keyes et al. [24] to assess mental health in a sample of Arabic psychologists aged 18–64 years. Our objective was to investigate the factor structure, internal consistency, and usefulness of the MHC-SF in determining functioning levels based on the Keyes model [19]. To the best of our knowledge, this study is one of the first to validate a measure of positive mental health, specifically in the Arabic population. Our findings provide initial evidence that MHC-SF is suitable for use with Arabic participants. These results are consistent with previous research on adults and older adults [21, 32], suggesting that this conceptualization of mental health applies from adolescence to adulthood. To conclude, we used the MHC-SF as a reliable questionnaire for assessing general mental well-being in an Arabic sample.

In conclusion, there is a potential for future research. Participants from both countries understood and interpreted the MHC-SF items in a similar and comparable style, which allows prospective studies to investigate students'mental health. However, the present research demonstrates the need for further investigation on whether the findings of the existing latent structure are replicable in different samples and countries.

## Strengths and Limitations

Our study had several strengths: incorporating input from focus groups allowed us to make necessary linguistic adjustments, which were generally well-received by the informants, who expressed a strong consensus regarding the relevance of the 14 items of the MHC-SF and the six items of the SVS. Another strength of our study is that the primary analyses were conducted using a sample from two Arab countries.

Our research has some limitations that should be acknowledged. First, the cross-sectional nature of the study limits the ability to draw causal conclusions. Second, the sample comprised primarily psychology students, which may reduce generalizability to other educational or clinical populations. Consequently, the psychometric characteristics of the MHC-SF for adolescents remain unclear.

Additionally, our research focused on the general population, and the factor structure may differ among clinical populations. Future research should aim to include adolescents from clinical populations. Furthermore, there is a need for psychometric evaluation of the MHC-SF among adults and the elderly in Arabic countries.

## Declarations

### Data Access

The authors do not wish to share the data, as that may breach participant confidentiality.
